# Overview of the DREADDs chemogenetics and the patent landscape of their actuators

**DOI:** 10.1007/s00044-026-03585-8

**Published:** 2026-08-03

**Authors:** Subhradeep Dutta, Malliga R. Iyer

**Affiliations:** Section on Medicinal Chemistry, National Institute on Alcohol Abuse and Alcoholism, National Institutes of Health, 5625 Fishers Lane, Rockville, MD 20852 USA

**Keywords:** Chemogenetics, DREADDs, GPCRs, Neural Circuits, Clozapine N-oxide, Neuromodulation

## Abstract

The ability to manipulate specific neural populations with high temporal and spatial precision has revolutionized our understanding of brain-behavior relationships. Among the most versatile tools in the chemogenetic arsenal are Designer Receptors Exclusively Activated by Designer Drugs (DREADDs). By introducing engineered proteins that remain inert to endogenous neurotransmitters but react potently to synthetic actuators, chemogenetics provides unparalleled control over neural circuits in vivo. With the field evolving, new designer ligands and designer receptors have been reported. This review provides an update on the latest advances in DREADD chemogenetics, focusing on the development of next-generation ligands and the patent landscape innovations driving the next era of precision neuromodulation.

**Figure:**
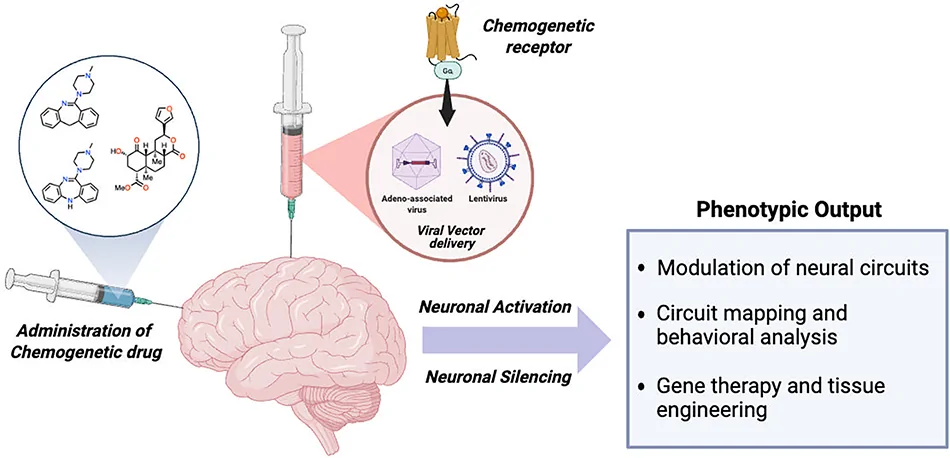

## Introduction

DREADDs (Designer Receptors Exclusively Activated by Designer Drugs) are engineered G protein-coupled receptors (GPCRs) that are unresponsive to endogenous ligands but can be activated by varying concentrations of an exogenous inert drug. These chemogenetic tools are used to selectively activate or inhibit neurons, thus allowing for the targeted modulation of neurocircuits in a way that is unrivalled by conventional drugs which may have unwanted effects due to their actions both in nontarget cells and brain regions, and on peripheral tissues. DREADDs have become a crucial tool for elucidating cells, circuits that govern a diverse range of phenotypes, including sleep, neuroinflammation, pain, motor activity, goal-directed and addiction behaviors along with metabolic dysfunction [1–4]. The emergence of DREADDS in the early 2000s, have since led to extensive acceptance by the neuroscientists, with numerous studies utilizing them across a broad range of research fields—from fundamental neuronal circuit mapping to modeling disease pathways. This growing use highlights both their versatility as experimental tools and the increasing interest in chemogenetics as a platform for developing next-generation therapies.

The first generation of engineered receptors were dubbed `genetically engineered receptors’ and could respond to otherwise inert compounds while lacking responsiveness to all known endogenous ligands [5]. The second generation were called RASSLs (Receptors Activated Solely by Synthetic Ligands) and were based on the κ-opioid receptor [6]. However, these engineered receptors had either low affinity towards their synthetic ligands or residual affinity for endogenous ligands along with limiting pharmacokinetic properties thus impeding their use. Subsequent development efforts centered on modifying muscarinic receptor backbones, resulted in several widely adopted designer receptors: hM3Dq, a G_q_-coupled excitatory receptor; hM4Di, a G_i_-coupled inhibitory receptor; and hM3Ds, a G_s_-coupled receptor that enhances cyclic Adenosine Monophosphate (cAMP) production [7]. These engineered variants are unresponsive to endogenous acetylcholine but exhibit high sensitivity to synthetic ligands, enabling precise control of downstream signaling pathways.

**Clozapine** ***N*-oxide** [CNO] **1** is the most widely used, systemically administered ligand to activate DREADDs, particularly hM4Di [1]. This selective activation was achieved through targeted mutations in the ligand-binding domain, allowing the receptors to interact with synthetic compounds like **1** [CNO] while eliminating responsiveness to native neurotransmitters such as acetylcholine (ACh) [5]. Importantly, these structural changes preserved the receptors’ ability to activate intracellular signaling upon ligand binding, while ensuring they remain inert under normal physiological conditions. This breakthrough paved the way for cell type–specific pharmacological modulation with unprecedented precision.

**1** [CNO] has a low affinity for endogenous receptors but can activate hM4Di with high specificity and potency, and is orally bioavailable, readily entering the brain. However, this prototypical DREADD agonist suffers from sluggish kinetics, poor brain penetrance and is metabolically degraded into the antipsychotic drug clozapine, which in the brain is an antagonist at dopamine D_2_, dopamine D_4_ and serotonin 2 A receptors and via poorly understood peripheral actions can trigger neutropenia and agranulocytosis [8–10]. In addition, there is limited information available on the off-target effects of the current alternative DREADD ligands that include **5** [olanzapine] and **2** [C21], although **5** [olanzapine] is like clozapine also an antipsychotic drug with off-target effects including weight gain [11]. Therefore, the advancement of the translational potential of this powerful chemogenetic technology to further its use in clinical settings necessitates understanding the off-target effects of these ligands to design novel, potent, and selective DREADD modulators that are also orally bioavailable with minimal off-target effects.

DREADDs hold significant potential for modeling and studying neurological disorders [4, 12]. While the promise of DREADDs is still expanding, their successful application relies on optimal delivery vehicles [2, 3]. Critically for their experimental usage, and for their many potential clinical applications, DREADDs packaged into Adenovirus Associated Virus (AAVs), can be delivered to specific brain regions via injection, thereby producing on-target effects but also off-target effects via transfection of cells in those regions to whatever extent the AAV strains are not cell specific. To develop treatments without off-target effects, different AAV serotypes that have distinct affinities for specific cell types are used, making them suitable for targeting particular cell populations. Cell type specificity of DREADD expression can be further enhanced via incorporating cell specific promoters into the DREADD construct. Recent developments in AAVs now allow for strategies to provide specific and efficient gene delivery to a targeted neuronal population by optimizing recombinant AAV genomic entities [13]. Size-reduced DREADD derivatives miniD_q_ and miniD_i_, which inherit the basic receptor properties from the G_q_-coupled excitatory receptor hM3D_q_ and the G_i_-coupled inhibitory receptor hM4D_i_, being approximately 30% smaller in size have been introduced to accommodate adeno-associated virus (AAV) deliveries for multiplexed interrogation [14]. While comprehensive reviews of the DREADDs and its application in neurosciences have appeared in the literature [2–4, 12], this review will focus on the state of the art in the field and the current intellectual property (IP) landscape of the ligands studied in activating or inhibiting DREADDS. Additionally, while chemogenetics has largely been focused on DREADDs, other chemogenetics-based techniques in this context, include chemically induced dimerization (CID), chemically induced transcription, and related [15] chemically regulated systems for protein stability or degradation. CID systems use small molecules to bring two engineered proteins together, while chemically induced transcription uses a small molecule to switch gene expression on or off. These techniques are covered in an excellent review by Kiyonaka et al. [15].

## Role of DREADDs in diseases

Since their development, DREADDs (Designer Receptors Exclusively Activated by Designer Drugs) have become indispensable tools in neuroscience, enabling precise manipulation of neural circuits to probe their roles in behavior and disease. Recent applications of DREADD technology have illuminated GPCR signaling pathways that are implicated in obesity, eating disorders, and metabolic syndrome disorders [1, 2, 12]. The capacity to selectively modulate neuronal activity in vivo has allowed for chemogenetics as a transformative approach in both basic research and translational modeling. A recent review delineated a comprehensive overview of the various classes of DREADDs, including optimal ligand dosing and examined the recent applications of DREADDs in psychiatry particularly around brain circuitry involved in anxiety [12].

### Neurological and neurodevelopmental disorders

DREADDs have been extensively used to model and manipulate the dopaminergic circuitry affected in Parkinson’s disease (PD) [3, 16–20]. Activation of dopamine D_1_ receptor–expressing neurons via G_q_-coupled DREADDs (e.g., hM3Dq) in PD models has led to significant improvements in motor function [21]. Moreover, long-term studies using G_s_-coupled humanized DREADDs have demonstrated sustained therapeutic effects, providing a framework for simulating clinical treatment regimens [22]. Targeted inhibition of hyperexcitable neuronal populations with G_i_-coupled DREADDs (e.g., hM4Di) has shown promise in reducing seizure frequency and severity in epilepsy models [23]. These interventions offer an advantage over traditional anticonvulsants by enabling circuit-specific modulation with minimal systemic side effects. DREADD-based modulation of limbic circuits, including the amygdala, hippocampus, and prefrontal cortex, has revealed how disruptions in excitatory-inhibitory balance contribute to mood disorders [3, 24]. Chemogenetic activation or suppression of specific cell types has been used to reverse behavioral phenotypes associated with depression and anxiety in rodent models [25–27]. Using DREADDs, researchers have modeled dysregulated cortical and subcortical connectivity hypothesized in schizophrenia [3, 28]. Manipulating pyramidal neuron activity or interneuron populations has helped uncover mechanisms underlying cognitive dysfunction [29], hallucination-like behaviors [30, 31], and social withdrawal [32]. DREADDs have been used to study neural circuit dysfunctions related to ASD[3], particularly in the context of social behavior [33–35], sensory processing [36], and repetitive actions [37]. By targeting key regions such as the medial prefrontal cortex and striatum, chemogenetics has provided insight into how abnormal connectivity patterns give rise to core symptoms.

One of the earliest applications of DREADDs has been in circuit mapping and behavioral analysis. By allowing for the targeted activation or silencing of specific neuronal populations, DREADDs enable to establish causal links between neural activity and behavioral outcomes. This methodology has been widely applied across diverse experimental paradigms, including studies of anxiety [38], fear conditioning [39], reward [40], feeding [41, 42], and sleep–wake regulation [43, 44], These investigations have illuminated the contributions of distinct neuronal subtypes and projection pathways to complex behaviors—insights that were previously out of reach using traditional pharmacological based approaches. These preclinical studies thus highlight the versatility of chemogenetics in modeling disease mechanisms and testing the effects of circuit-specific interventions.

### Peripheral diseases and metabolic disorders

DREADDs have emerged as powerful tools for dissecting the complex neurobiological and peripheral mechanisms underlying energy balance, glucose homeostasis, thermogenesis, lipid metabolism, and feeding behavior. By enabling precise, reversible, and cell-specific modulation of neuronal or non-neuronal activity, DREADDs offer unique advantages over traditional genetic knockouts, optogenetics, or pharmacology.

Emerging studies have employed DREADDs to manipulate cardiac rhythm [45–48] and gastrointestinal motility [49], paving the way for non-invasive, circuit-specific modulation of peripheral organ systems. The hypothalamus is a key regulator of energy homeostasis, integrating hormonal, neural, and nutrient signals to coordinate feeding and energy expenditure [50]. DREADDs have been instrumental in studying hypothalamic circuits, for e.g. AgRP (Agouti-related peptide) neurons: Activation of AgRP neurons via hM3Dq induces hyperphagia (increased food intake), while inhibition via hM4Di suppresses feeding, demonstrating their orexigenic role [1]. Activation of POMC (pro-opiomelanocortin) neurons reduces food intake and enhances energy expenditure [51]. Chemogenetic manipulation of VMH (ventromedial hypothalamus) and PVN (paraventricular nucleus) has helped clarify their roles in satiety, glucose regulation, and sympathetic outflow to peripheral organs. DREADDs have been used to study how central neurons respond to hormones like leptin, ghrelin, and insulin. Activating insulin-sensitive neurons in the arcuate nucleus (ARC) has been shown to modulate glucose tolerance and hepatic glucose production. Further chemogenetic activation or inhibition of specific neuronal populations using DREADDs enabled the investigation of how autonomic nervous system output from the central nervous system (CNS) influences pancreatic insulin secretion and hepatic gluconeogenesis [52]. Activation of VMH neurons via G_q_-DREADDs showed an increase in sympathetic output and improvement of glucose uptake in peripheral tissues [53, 54]. DREADDs are increasingly applied to peripheral tissues, offering new ways to study metabolic regulation outside the brain [55, 56]. DREADDs have played an important role in understanding adipose tissue thermogenesis [57, 58]. Activation of sympathetic efferents using DREADDs has been shown to increase BAT (Brown adipose tissue) thermogenesis and energy expenditure [59, 60]. Modulating innervation to WAT (White adipose tissue) using DREADDs has illuminated lipid mobilization and browning processes [61]. DREADDs have been used to manipulate hepatic vagal innervation, affecting gluconeogenesis, glycogen storage, and lipid metabolism [62]. Peripheral DREADD expression in hepatocytes (through viral vectors) has allowed direct modulation of metabolic enzyme activity [52, 63]. Chemogenetic tools have been applied to study the regulation of β-cell insulin secretion [64–66] and α-cell glucagon release [67, 68], to delineate brain-pancreas communication. In the context of skeletal muscle signaling, DREADDs have been used to explore how motor neuron activity or muscle innervation affects glucose uptake and fatty acid oxidation [69].

While the central nervous system has been the primary focus of DREADD research, recent advances have expanded their application to peripheral tissues. Development of the hydroxycarboxylic acid DREADD (HCAD), based on the HCA2 receptor, which is primarily expressed in the immune system with limited CNS distribution [70]. HCAD enables selective modulation of immune and metabolic pathways without off-target central effects [70, 71]. Parallel efforts are exploring chemogenetic control in peripheral organs such as the liver [63, 72], heart [46, 73], and gastrointestinal tract [49, 74], signaling a broader shift toward system-wide neuromodulation.

These emerging peripheral applications underscore the expanding relevance of DREADDs beyond neuroscience. As delivery methods improve and receptor variants are tailored for specific tissues, chemogenetics holds growing potential in fields such as immunology, endocrinology, and cardiology—marking a significant evolution from its neurological origins.

## DREADDS and its chemical actuators

**Fig. 1:**
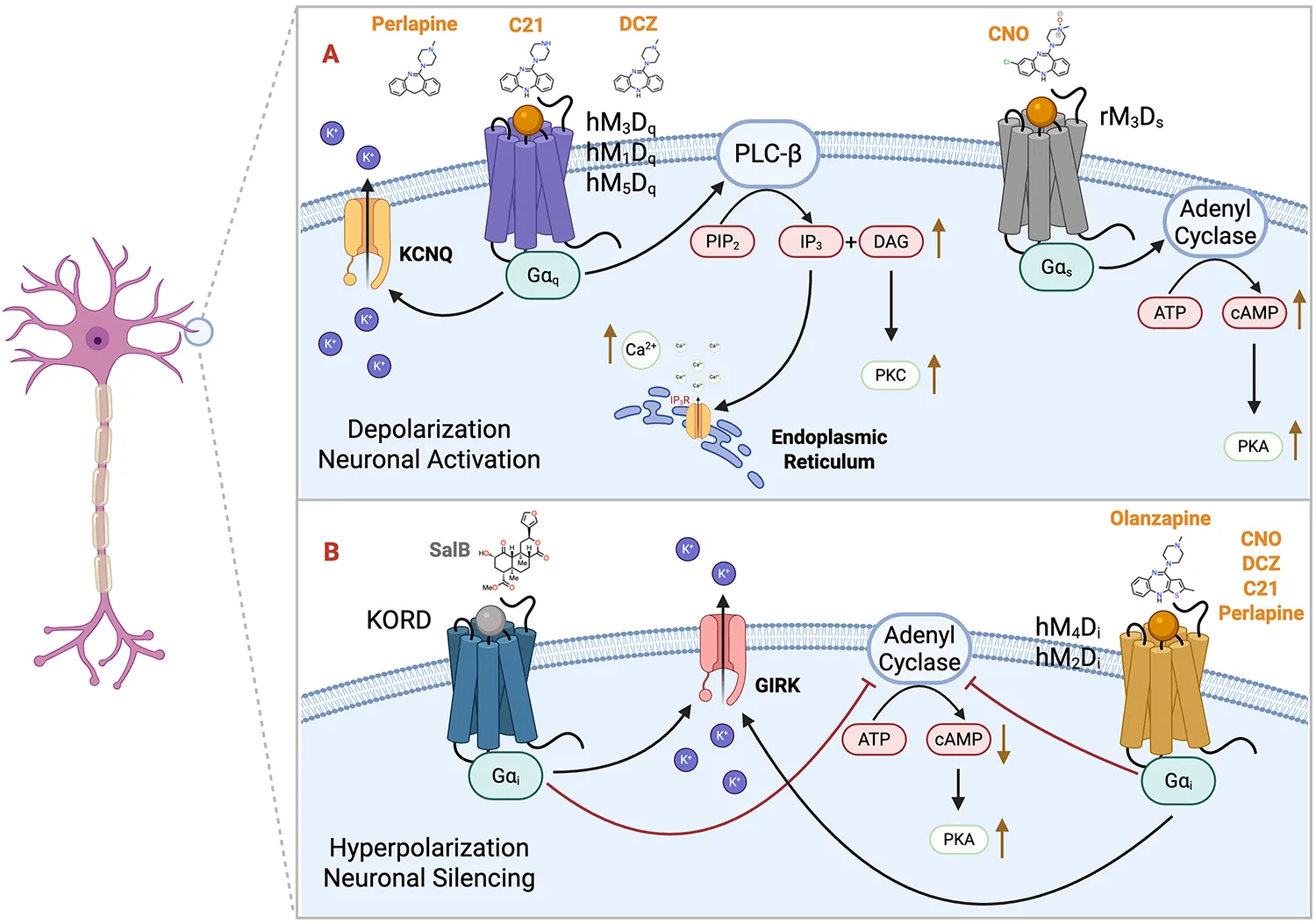
Overview of signaling pathways and mechanism of action of various DREADDs. Designer Receptor Exclusively Activated by Designer Drugs (DREADDs) are classified into two categories excitatory and inhibitory modulators. **A** hM1Dq, hM3Dq and hM5Dq are coupled to the G_q_ signaling pathway. Ligand binding activates phospholipase C, which then catalyzes the hydrolysis of phosphatidylinositol 4,5-bisphosphate (PIP_2_) to inositol 1,4,5-trisphosphate (IP_3_) and 1,2-diacylglycerol (DAG). Both IP_3_ and DAG function as secondary messengers: IP_3_ facilitates the release of Ca^2+^ from the endoplasmic reticulum and DAG activates protein kinase C (PKC). rM3Ds is coupled to the G_s_ signaling pathway and upon ligand binding activates the adenylyl cyclase (AC) which in turn increases the cyclic adenosine monophosphate (cAMP) levels thereby enhancing protein kinase A (PKA) levels. Both G_q_ and G_s_ serve as excitatory modulators and their activation subsequently leads to the opening of Potassium Voltage-Gated Channel Subfamily Q Member (KCNQ channels) leading to depolarization and enhanced neuronal signaling. **B** hM2Di, hM4Di and KORD are coupled to the G_i_ signaling pathway and upon ligand binding inhibits AC activity, reducing downstream cAMP levels and induces PKA. Activation of inhibitory G_i_-DREADDs opens up G protein-coupled inwardly rectifying potassium channels (GIRKs) thereby leading to hyperpolarization and ultimately inhibition of neuronal signaling. Figure created with BioRender.com

When DREADDs were first introduced, three G_q_-coupled variants—hM1Dq, hM3Dq, and hM5Dq—were developed, each derived from a different human muscarinic receptor. These Gq-DREADDs are all activated by nanomolar concentrations of **1** [CNO], leading to increased intracellular calcium signaling. While any of the three can function as excitatory DREADDs, hM3Dq is most commonly employed in neuroscience research (Fig. 1).

G_i_-DREADDs are engineered chemogenetic receptors that, when activated, inhibit neuronal activity by coupling to inhibitory G_i/o_-protein signaling pathways. The most recognized G_i_-DREADDs are hM2Di and hM4Di, both derived from human muscarinic acetylcholine receptors (M2 and M4) and selectively activated by designer ligands like **1** [CNO].

The principal G_s_-DREADD in use is rM3D_s_, which was engineered by combining structural elements of a rat muscarinic M3 receptor and a turkey β-adrenergic receptor to confer selective coupling to G_s_ proteins. Upon activation by designer drugs such as **1** [CNO], G_s_-DREADDs stimulate adenylate cyclase activity, leading to increased intracellular cAMP concentrations and the activation of protein kinase A (PKA) signaling [75]. Since G_αolf_ is the predominant G_s_-like G_α_ protein in certain brain areas, it was important to assess whether GsD could activate G_αolf_. Studies have confirmed that GsD robustly couples to G_αolf_ both in vitro and in vivo, exhibiting only minimal constitutive activity in G_αolf_-mediated signaling under these conditions [76]. GsD has been leveraged to investigate the contributions of G_s_-like pathways to various behaviors, such as ethanol intake [77], reward [78], locomotor sensitization [75], and circadian rhythms [79]. G_s_-DREADDs offer powerful, reversible control over cAMP signaling and neuronal activity, but their application is currently more restricted than other DREADD variants.

**Fig. 2:**
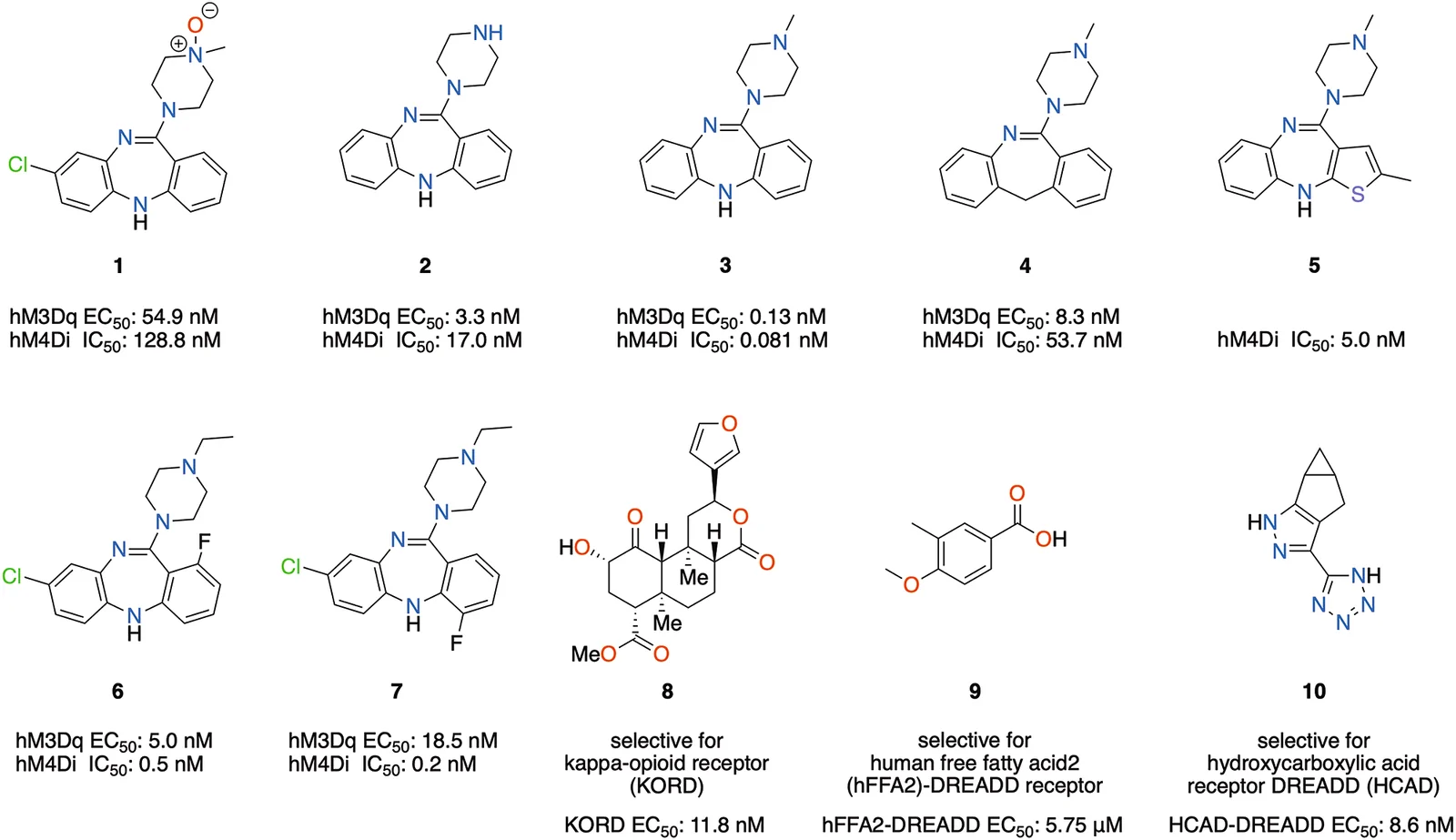
Chemical structures of reported DREADDs activators

DREADD actuators are shown below in Fig. 2 along with their chemical structures.

As alluded to previously, **1** [CNO], the inactive metabolite of the atypical antipsychotic drug clozapine (CZP), was the first synthetic and most widely studied “designer drug” in the context of DREADDs [12, 80–82]. While initially thought to be a selective activator of mutated G protein-coupled receptors such as hM3Dq, hM4Di, hM2Di, and hM5Dq, emerging evidence now show that **1** [CNO] is actively exported from the CNS and back-converted to CZP, which crosses the blood-brain barrier and subsequently acts as the ligand activating the DREADDs [10, 83]. Due to the safety concerns associated, **1** [CNO] has not been approved for clinical use (Fig. 2).

**2** [C21] (Compound 21) is a potent and selective agonist that activates muscarinic-based DREADDs including hM3Dq, hM1Dq, and hM4Di [84, 85]. It demonstrates high bioavailability, excellent brain penetration, and serves as an alternative to **1** [CNO] particularly where metabolic instability is a concern, ensuring more specific chemogenetic control during in vivo studies.

**3** [DCZ] (Deschloroclozapine) is a next-generation chemogenetic agonist designed to selectively activate DREADDs (Designer Receptors Exclusively Activated by Designer Drugs), particularly hM3Dq and hM4Di, which are engineered muscarinic receptors used to modulate neural activity [83, 86]. It represents a major improvement over earlier ligands such as **1** [CNO] and **2** [C21] in terms of potency, pharmacokinetics, and brain specificity. **3** [DCZ] is extremely potent, with reported EC_50_ of 0.13 nM for excitatory hM3Dq DREADD and 0.081 nM for hM4Di inhibitory DREADD. It displays about100-fold higher potency than **1** [CNO] and 40–60-fold higher than **2** [C21]. **3** [DCZ] was shown to be highly selective for DREADDs, showing minimal interaction with over 300 other GPCRs and ion channels. Its off-target binding is greatly reduced compared to clozapine.

**4** [Perlapine] is a potent chemogenetic actuator that functions as an agonist for hM3Dq, hM1Dq, and hM4Di [84, 85]. Originally synthesized as a hypnotic and sedative drug for treating insomnia in Japan, it was later identified through receptor screening as a non-CNO DREADD agonist with high potency and distinct pharmacological characteristics. **4** [Perlapine] selectively activates hM3Dq, hM1Dq, and hM4Di DREADDs, with pEC₅₀ values of 8.08, 8.38, and 7.27, respectively. It exhibits > 10,000-fold higher affinity for hM3Dq over the wild-type hM3 receptor and relatively low affinity for unmodified hM1 and hM4, showing it preferentially activates designer receptors over native muscarinic GPCRs. Unlike **1** [CNO] which back metabolize to clozapine and leads to confounding behavioral results upon DREADD activation, perlapine is an approved drug with a known safety profile which allows for it to be utilized in vivo modulation of neuronal activity. While generally selective, perlapine exhibits measurable affinity for serotonin (5-HT₂A, 5-HT₂C, 5-HT₇), dopamine D₁, and histamine H₁ receptors, explaining its sedative and REM-suppressing effects.

**5** [Olanzapine], an atypical antipsychotic has been repurposed as an actuator for human muscarinic-based inhibitory DREADDs. It is a full, high-potency agonist at hM4Di receptor (EC_50_= 5 ± 2 nM). Additionally, low systemic doses of **5** [olanzapine] (e.g., 0.1 mg/kg i.p. in rodents) has been shown to produce desired behavioral effects in DREADD-expressing animals in vivo [87]. Unlike **1** [CNO], **5** [olanzapine] is widely available in clinical formulations and already been approved for human use, thus making it an attractive tool for potential clinical chemogenetic.

**6** [JHU37152] and **7** [JHU37160] are synthetic chemogenetic ligands developed as high-potency, blood–brain barrier–penetrant agonists for DREADDs [88]. They were created to overcome the limitations of earlier ligands such as **1** [CNO], which had poor brain permeability and metabolic instability. Both **6** [JHU37152] and **7** [JHU37160] act as potent in vivo DREADD agonists for hM3Dq and hM4Di. They produce robust receptor occupancy in the brain and are not substrates for P-glycoprotein (P-gp), allowing effective brain entry. **6** [JHU37152] increases hM3Dq-stimulated locomotor activity and inhibits hM4Di-driven activity when expressed in dopaminergic neurons. **7** [JHU37160] causes similar DREADD activation but has been shown at high doses (e.g., 1 mg/kg) to induce anxiogenic effects in rats independent of DREADD expression, indicating off-target activity.

SalB DREADD is a chemogenetic system that uses **8** [SalB] (salvinorin B) as a selective ligand to control cell and circuit activity via a modified kappa-opioid receptor DREADD known as KORD (κ-opioid receptor DREADD) [89, 90]. *KORD-SalB system*, is a designer receptor system created from the κ-opioid receptor that has been mutated to respond exclusively to **8** [SalB], an otherwise pharmacologically inactive metabolite of salvinorin A, the psychoactive component of *Salvia divinorum*. SalB binds and activates the KORD receptor but has no notable effect on native kappa-opioid receptors. When activated, KORD engages the G_i_ signaling pathway, which inhibits neuronal activity by reducing cAMP levels and suppressing action potential firing. **8** [SalB] readily crosses the blood-brain barrier and is pharmacologically inert at physiological receptors, enabling in vivo studies. **8** [SalB] represent non-nitrogenous scaffolds adapted from KOR agonists. Mutation D138^3.32 N^ in the receptor allows selective activation by salvinorin analogues such as momSalB, demonstrating the extension of DREADD design beyond muscarinic templates.

**9** [MOMBA] (4-methoxy-3-methylbenzoic acid) is a small-molecule ligand designed to selectively activate the hFFA2-DREADD (engineered version of the human free fatty acid receptor 2). It is a highly selective orthosteric agonist with no detectable activity at wild-type human or mouse short chain fatty acid (FFA2 or at FFA3) receptors [91]. Functionally, **9** [MOMBA] is more potent compared to sorbic acid (4-hexadienoic acid) [pEC50 = 5.24 ± 0.16 Vs pEC50 = 4.77 ± 0.15, respectively]. Furthermore, oral administration of **9** [MOMBA] in hFFA2-DREADD knock-in mice has been shown to reduce gut transit and promote GLP-1 release from colonic crypts, thus making it a valid tool compound to probe FFA2 physiology.

**10** [FCH-2296413] was developed as a chemogenetic actuator to selectively activate G_i_-coupled HCAD (hydroxycarboxylic acid receptor DREADD). HCAD is engineered from the native HCA2 receptor, which is minimally expressed in the brain or other neuronal tissues. **10** [FCH-2296413] demonstrated low brain penetration and a clean off-target profile in broad screening (e.g., across hundreds of GPCRs), supporting its use as a selective chemogenetic tool [70]. Additionally high-resolution structural data including cryogenic electron microscopy (cryo-EM) structure of HCAD-G_i_ complex with **10** [FCH-2296413] now sheds light into the key interactions involved in the ligand recognition pocket and provide a foundation for the rational design of peripheral chemogenetic systems.

## DREADD delivery methods

In order to design a DREADD-based therapy, it is necessary to first evaluate the viral vector delivery methods utilized in the literature to deliver these chemogenetic tools. That the viral vectors do not contribute to added deleterious effects acutely or long term is an important safety consideration [92]. This section evaluates each of the prominent strategies currently utilized for DREADD delivery, discussing their respective advantages and limitations. We focus on adeno-associated virus (AAV)-based and lentivirus-based systems, and the manipulation of these through cell-type specific promoters and pseudotyping.

### AAV-virus DREADD delivery

Adeno-associated viruses (AAVs) are small, single-stranded DNA viruses that have been used as gene delivery vectors since the mid-1980s [93, 94]. Their favorable safety profile, low immunogenicity, and capacity for long-term transgene expression have established AAVs as a leading platform for gene therapy, including applications targeting the CNS. Importantly, AAV-mediated transgene expression can be confined to distinct cell populations through the combined use of cell-type specific promoters and tailored capsids, thereby enhancing precision and minimizing off-target effects [95–99]. The clinical success of AAV-based gene therapies, such as Zolgensma (approved for the treatment of spinal muscular atrophy), underscores its translational potential. AAVs have been widely adopted to deliver DREADDs to neurons, primarily in preclinical studies aimed at understanding neural circuits, behavior patterns, and disease pathways. Evidence from these studies have now bolstered the feasibility of DREADD-based neuromodulation and lay the foundational groundwork for the rational design of future therapeutic interventions. While AAVs have strong foundation, vigilance is needed by utilizing long term monitoring in AAV-based therapy is warranted [100].

#### Enhancing accuracy of DREADD expression using cell-type specific promoters [101–103]

One of the key determinants of accurate DREADD expression is promoter selection. In this context, the human synapsin 1 gene (hSyn) promoter has been widely used to drive specific neuronal expression and has demonstrated reliable targeting across species, including non-human primates (NHPs) [104]. For instance, a study conducted by Deffains et al., utilizing the hSyn promoter have highlighted the ability to modulate external globus pallidus neurons in NHPs, suggesting potential relevance of DREADDs to improve disease phenotypes with origins in basal ganglia such as Parkinson’s disease[105]. Similarly, the use of CaMKIIα promoter enables preferential expression in excitatory neurons of the neocortex and hippocampus [106]. In a study done by Chiou et al., CaMKII promoter was utilized both to study neural mechanisms underlying behavior and to improve disease-associated phenotypes, including chronic pain in mouse model of bone cancer [107].

A major impediment to the use of different promoters as part of the AAV-based strategy is their limited packaging capacity, which constrains promoter size and transgene complexity [108, 109]. To address this, truncated promoter sequence such as the gfaABC1D fragment of the GFAP glial promoter, which enables astrocyte-specific DREADD expression, has been developed [110]. Route of administration represents an additional translational consideration. Majority of the studies involving DREADDs have relied on local intracranial AAV injections, which are invasive and less suitable for clinical application. Consequently, limited data is available on the efficiency and specificity of DREADD expression following systemic administration. In addition, evidence from a study done by Upright et al. on NHPs suggests that relatively low levels of neuronal transduction may be sufficient to induce significant behavioral effects and hence achieve a desired behavioral change [111].

#### Enhancing tropism through AAV Serotypes [13, 95]

In addition to promoter selection, choice of AAV serotype plays a crucial role in defining viral tropism and transduction efficiency [112, 113]. For instance, certain serotypes, particularly AAV9 are capable of crossing the blood-brain barrier (BBB), enabling systemic delivery to the CNS [114]. However, pre-existing neutralizing antibodies in certain demography’s represent a significant translational barrier. To increase transduction efficiency and reduce immunogenicity, pseudotyped vectors and engineered capsids such as PHP.B and its derivative PHP.eB have been developed. Morabito et al. utilized AAV-PHP.B capsids to achieve precise, functional DREADD expression in defined neuronal populations, with chemogenetic inhibition of parvalbumin-positive (PV+) interneurons revealing their role in seizure susceptibility [115]. These findings demonstrate the utility of engineered AAV capsids for DREADD delivery in circuit-specific studies, although species specific constraints limit their suitability for systemic administration [116, 117].

### Lentiviral DREADDs delivery [118]

Although adeno-associated viral (AAV) vectors remain the most widely used system for the delivery of DREADDs to the CNS, lentiviral vectors provide a complementary approach with several distinct advantages. Lentiviruses are retroviruses with an RNA genome that is reverse-transcribed into double-stranded DNA and integrated into the host genome following cellular entry [119]. Since their early development as gene delivery tools, lentiviral system has undergone extensive refinement particularly in terms of safety [120–122]. Modern third generation lentiviral vectors lack accessory virulence genes, employ split-genome designs across multiple plasmids, and often incorporate self-inactivating long terminal repeats (LTRs), thereby significantly reducing the risk of replication-competent virus formation and unintended transcriptional activation [123–127].

In comparison to AAVs, lentiviral vectors offer larger packaging capacity, enabling the use of larger promoters and complex genetic cassettes that can enhance expression control and transduction efficiency [128]. Lentiviruses are most commonly pseudotyped with the vesicular stomatitis virus G (VSV-G) envelop, which confers broad cellular tropism and allows efficient transduction of a wide range of cell types [124, 129–131]. Similar to AAV vectors, cell type specificity in lentiviral DREADD delivery is largely governed by promoter selection. Additionally, emerging work on alternate viral envelopes or novel chimeric envelopes continues to improve target precision and hence suitability for DREADD delivery.

#### Promoters for lentiviral DREADD expression

Promoters previously validated in AAV-based DREADD studies, including human-synapsin (hSyn) and CaMKII, have proven to be effective in lentiviral systems as well. For instance, Mahler et al. have successfully utilized synapsin-driven lentiviral delivery of the inhibitory DREADD hM4Di to produce spatially restricted neuronal expression and robust functional inhibition following **1** [CNO] administration [132]. Additionally, evidence from their study supports anterograde trafficking of DREADDs, facilitating modulation of interconnected neural circuits than isolated neuronal population.

Similarly, CaMKII-driven lentiviral expression has been used to selectively target excitatory neurons. In a study done by Caracciolo et al. on post-stroke motor recovery, CaMKII-driven lentiviral co-expressing hM4Di and CREB enabled precise and reversible inhibition of targeted excitatory neurons, resulting in impaired motor recovery following chemogenetic activation [133]. Lentiviral vector was chosen over AAV to achieve more localized transgene expression.

#### Engineering lentiviral envelopes for enhanced targeting

VSV-G pseudotyping has been known to provide broad tropism, however it does not efficiently facilitate retrograde transport, thereby limiting its applicability for circuit level studies [134, 135]. To address this impediment, alternative lentiviral envelopes have been developed, including chimeric rabies virus glycoproteins such as FuG-B2, which enhances retrograde gene transfer in the CNS [135–138]. In a recent study Hayashi et al. utilized FuG-B2-pseudotyped lentiviral vectors expressing hM4Di to selectively modulate neuronal activity and behavior in non-human primates (NHPs) [139]. This study demonstrates the potential of envelope-engineered lentiviruses for targeted neuronal circuit manipulation.

## Structural biology studies

**Fig. 3:**
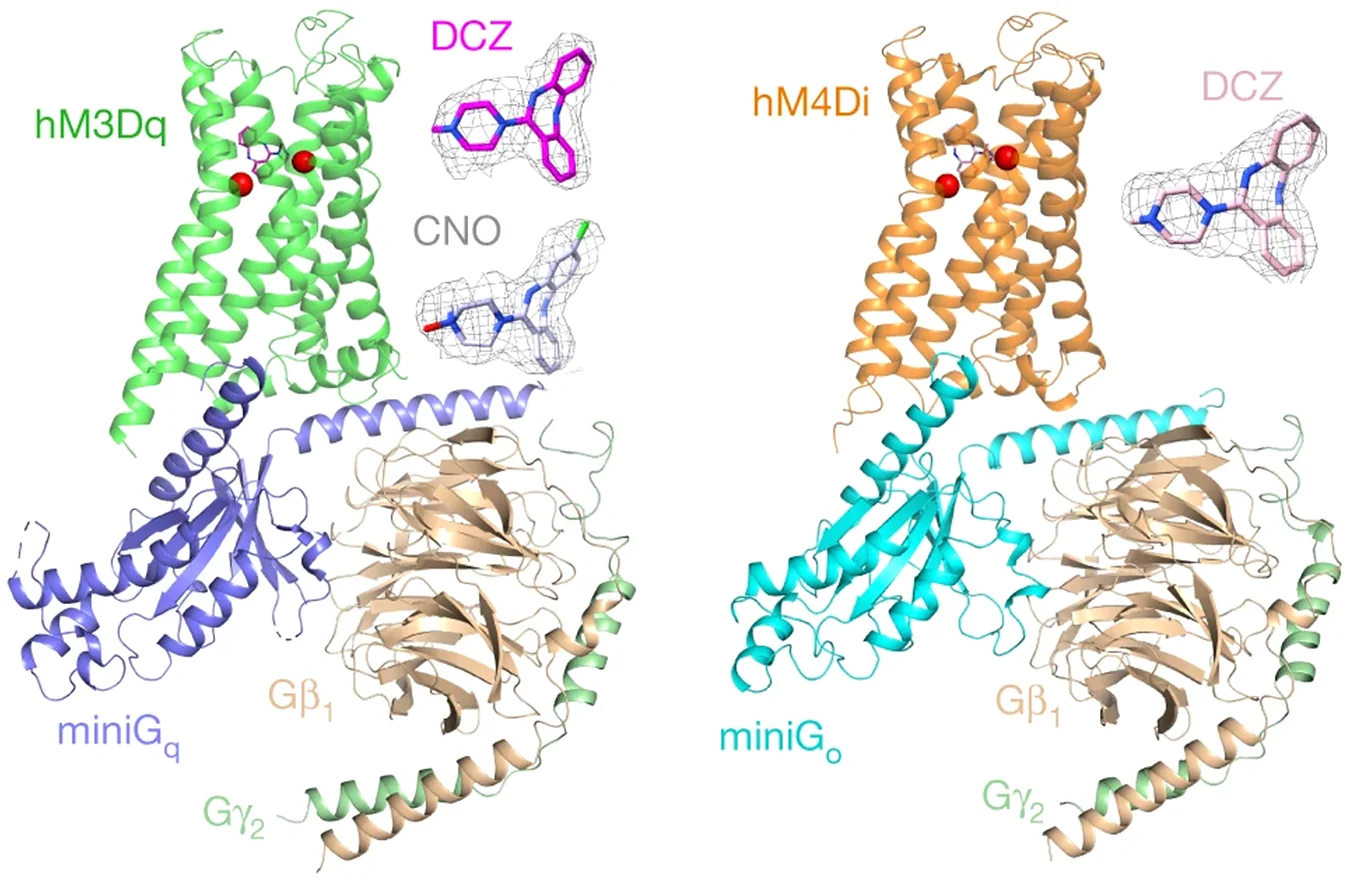
Overall structures of the DCZ-bound hM3Dq–miniG_q_ and hM4Di–miniG_o_ complex *8E9W* (*EMD-27966*), *8E9X*(*EMD-27967*),^140^ Reproduced with permission

The structural biology of DREADDs was an important milestone that revealed the molecular determinants of engineered GPCRs which are mutated versions of muscarinic acetylcholine receptors designed to be selectively activated by designer non endogenous synthetic ligands. Key structural insights were derived from high-resolution cryo-EM structures of DREADDs hM3Dq and hM4Di complexed with ligands like **3** [DCZ] and **1** [CNO] [140]. These structures showed ligand binding pockets distinct from wild-type receptors with mutations that reduce affinity for endogenous acetylcholine while increasing sensitivity to synthetic agonists (Fig. 3). Four high-resolution cryo-electron microscopy structures associated with DREADDs were recently reported (Fig. 3): an hM3Dq–miniG_q_ complex and an hM4Di–miniG_o_ complex, both bound to **3** [deschloroclozapine]; an hM3Dq–miniG_q_ complex bound to **1** [CNO]; and an hM3R–miniG_q_ complex bound to iperoxo [140]. Through complementary mutagenesis, functional assays, and computational simulations, these structures revealed critical insights into how DREADD chemogenetic actuators are recognized and uncover the molecular mechanisms underlying receptor activation. Mutations in residues like Asp^3.32^, Tyr^6.51^, and Asn^6.52^ reduce binding of acetylcholine while facilitating hydrophobic or π–π interactions specific to synthetic ligands. Structural comparisons highlighted key amino acid residues in the binding pocket responsible for ligand specificity and receptor activation mechanisms.

The receptors couple to specific G proteins (G_q_ for hM3Dq, G_i/o_ for hM4Di), and ligand binding triggers conformational changes that activate intracellular signaling pathways such as phospholipase C (PLC) activation and calcium release for G_q_-coupled DREADDs or inhibition of adenylate cyclase and activation of potassium channels for G_i_-coupled receptors. Ligand binding sites show altered conformation compared to wild-type, especially in TM4, TM5, and TM6 helices. Activation mechanism of the ligand involves allosteric changes propagating to G protein coupling interfaces, enabling selective downstream signaling.

Ligand-specific stabilization of transmembrane helical shifts (notably TM6 displacement in hM3Dq) drives selective G-protein engagement, defining downstream efficacy.

These high-resolution cryo-EM maps (∼2.7–3.2 Å) defined how the mutated residues in the orthosteric pocket shift the receptor’s conformational equilibrium toward ligand-specific activation. These advances clarified how synthetic ligands selectively activate DREADDs and facilitate structure-guided design of improved chemogenetic tools for precise neuronal and cellular control.

**Fig. 4:**
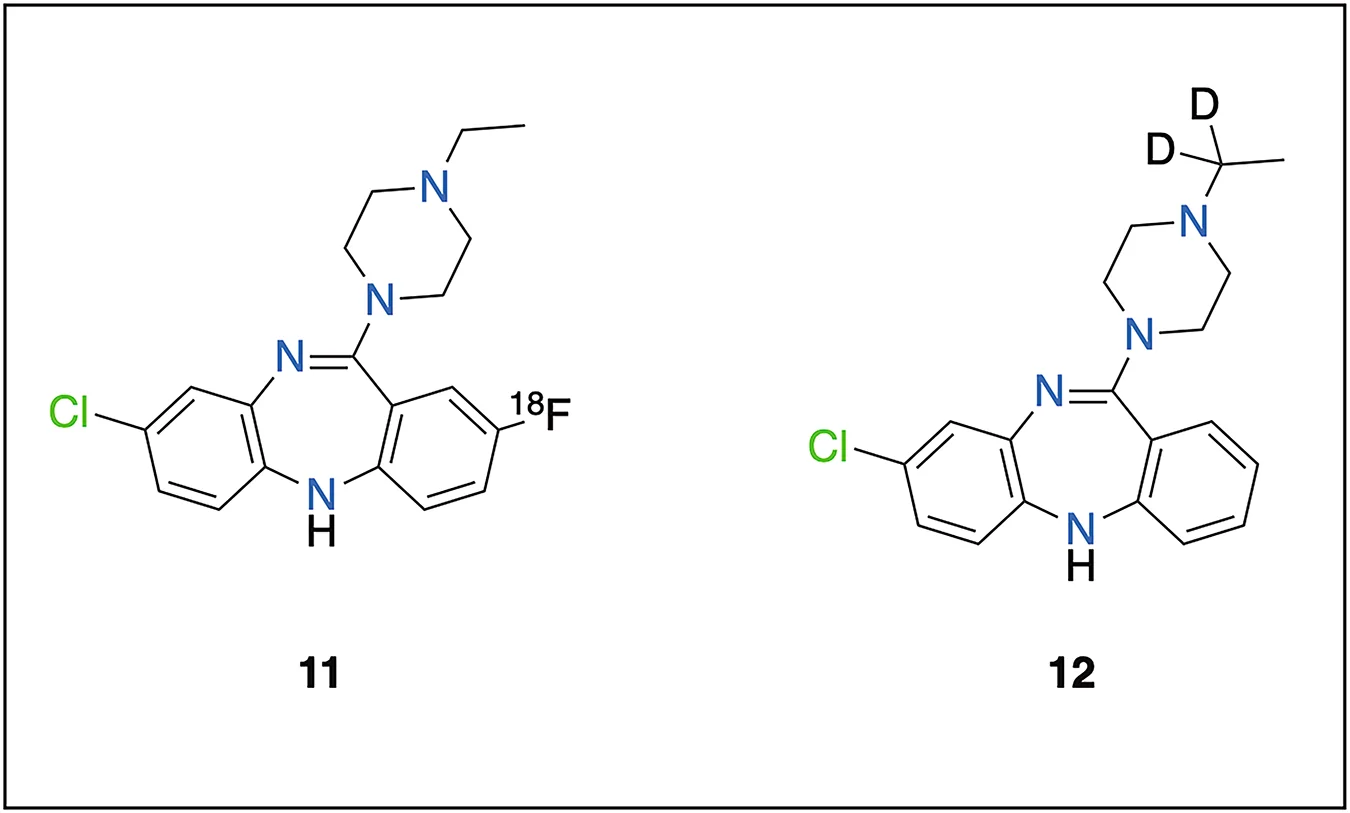
PET-compatible DREADD ligands: [^18^F]**11** and [^3^H]**12**

Structure-informed design has now guided the design of new DREADDs such as of peripherally restricted or multiplexed DREADDs, such as those based on hydroxycarboxylic acid receptors or κ-opioid receptors (KORDs) [70, 89, 90]. These were designed to minimize CNS penetration or to allow dual activation of distinct GPCR pathways in the same cell population. In parallel, the atomic structure of muscarinic M4 receptor (PDB 5DSG) further served as a template for designing PET-compatible DREADD ligands such as [^18^F]**11** and [^3^H]**12** (Fig. 4).

## Current IP related to DREADDs

In principle, DREADDs can be designed with any GPCRs through structure-guided optimization of native GPCR structures. Earlier work in the DREADDs largely involved open-source tools pioneered by Roth et al. [4, 85, 89, 90, 140]. As the field of GPCR DREADDs evolves, the translational pathway dictates current IP strategies involving the DREADDs. A brief overview of the patents in the field is outlined below.

### UCL Business Ltd

Inventors from UCL Business Ltd. disclosed a novel chemogenetic strategy for the treatment of seizures, particularly focal epilepsy (US10525103B2) [141]. This strategy employs viral-vector mediated delivery of DREADDs to selectively modulate the excitability of restricted neuronal population within the seizure focus only in the presence of an externally administered agonist. Inhibitory DREADD hM4Di was delivered utilizing a AAV5 vector under a CaMKII promoter to target excitatory cortical neurons. In vivo studies conducted in rat demonstrated a significant reduction in seizure frequency in two acute focal seizure models induced by pilocarpine and picrotoxin, and attenuated spontaneous epileptiform activity in the chronic tetanus toxin model of focal neocortical epilepsy. Furthermore, data from histological and behavioral studies indicate localized cortical expression, and no evident baseline motor deficit, while control experiments supported the specificity of the chemogenetic effect. Overall, the authors present a reversible, regionally targeted seizure inhibition as a potentially attractive alternative to permanent excitability-altering gene therapies and to optogenetic approaches that require implanted light-delivery hardware.

Kullman and colleagues at UCL Business Ltd. further described a new chemogenetic receptor class termed GRANPAs (G-protein coupled Receptors Activated by Non-Prescription Agents) (US20250032643A1) [142]. This was designed to overcome a key limitation of earlier DREADDs which is their dependence on prescription ligands such as clozapine or **5** [olanzapine]. Starting from the inhibitory muscarinic DREADD (hM4Di), the authors utilized structure-guided mutagenesis and functional screening to generate receptors with reduced responsiveness to endogenous ligand acetylcholine but activate upon binding to well-known antihistamine diphenhydramine. These optimized variants achieved markedly stronger diphenhydramine responses (functional potency in the low nanomolar range) as evidenced from arrestin, G_i_/cAMP and GIRK electrophysiological assays. Y113C+A203G+S85V+V120I+Y416F+L123T was identified as the preferred construct as it preserved agonist responsiveness while minimizing basal activity. Furthermore, several additional positions were identified that can further tune receptor pharmacology thereby expanding the receptor scope as well as the ligand scope that can activate those receptors with improved activity. Studies conducted in mice, using AAV9 delivery of the lead construct to substantia nigra showed that diphenhydramine could alter behavior in vivo, supporting the feasibility of reversible circuit control with an over the counter (OTC) drug.

### California Institute of Technology

Shapiro and colleagues at the California Institute of Technology (US11400168B2) revealed a non-invasive neuromodulation strategy combining focused ultrasound-induced, microbubble-assisted transient blood-brain barrier opening with systemic delivery of AAV vectors encoding chemogenetic receptors, followed by systemic administration of a chemogenetic ligand [143]. The platform was designed to integrate three levels of precision: spatial targeting through focused ultrasound, cell-type specificity through promoter choice or intersectional genetic design, and temporal control through ligand dosing. MRI-guided targeting of mouse hippocampus produced broad DREADD expression across dorsal and ventral hippocampal subfields, with a noticeably strong transduction in CA2, CA3, and dentate gyrus, while untargeted thalamus and peripheral dorsal root ganglia showed little to no detectable expression. Functional validation showed that excitatory hM3Dq increased c-Fos in targeted hippocampal neurons, whereas inhibitory hM4Di reduced contextual fear-memory formation after **1** [CNO] administration without altering open-field exploration or cue-associated freezing during training, supporting a circuit-selective effect rather than a non-specific motor or sensory deficit. Additionally, greater dorsal CA3 expression correlated with a larger reduction in fear recall, whereas post-FUS MRI enhancement did not predict final expression levels. Data from histology suggest generally acceptable tolerability in these experiments: most focused-ultrasound sites appeared normal, and the lesions observed at a minority of sites were substantially smaller than the tissue disruption associated with conventional intracranial needle injections. This multimodal framework offers potential therapeutic applications for a range of neurological and psychiatric disorders and could be an alternative to direct intracranial injection or chronically implanted neuromodulation hardware for achieving selective control over neural circuits.

### Coda Biotherapeutics, Inc

Greenberg and co-workers at Coda Biotherapeutics, Inc. reported a gene therapy-based neuromodulation platform for treating neurological disorders (US2018193414A1) [144]. The approach involves delivery of G protein-coupled receptor or ligand-gated ion channel selectively to a defined neuronal population, then activate or silence them using small-molecule ligands. While the authors propose broad applications across pain, epilepsy, satiety disorders, post-traumatic stress disorder (PTSD), gastroesophageal reflux disease (GERD), Parkinson’s disease, Alzheimer’s disease, movement disorders, and other neurological conditions, the patent work specifically focuses on pain management. The invention focuses on chemogenetic analgesia using an AAV-based vector to express an engineered human glycine receptor alpha-1 mutant, GlyR-M, in pain pathways. In particular, the patent emphasizes AAV6 and engineered AAV6 capsids, typically paired with the hSyn (human synapsin) promoter, to drive neuronal expression after intrathecal, intraganglionic, or intraspinal delivery. Studies done on Spared Nerve Injury (SNI) model in mice demonstrated robust transgene expression in dorsal horn and dorsal root ganglion neurons and a marked reversal of mechanical hypersensitivity after systemic ivermectin administration, with analgesic effects lasting roughly 7–10 days after a single dose and recurring after repeated dosing. Furthermore, trigeminal ganglion experiments in rats showed longer thermal withdrawal latencies after ivermectin administration, consistent with reduced thermal pain sensitivity. Compared to conventional pharmacological treatments, this work provides enhanced spatiotemporal precision and sustained therapeutic control, potentially improving both safety and efficacy in pain management.

### Rutgers University

Bowrey and Aston-Jones at Rutgers University proposed a chemogenetic strategy for modulating central noradrenergic circuits, particularly the locus coeruleus by targeting retinal cells (US2023174613A1) [145]. Instead of introducing DREADDs directly into the brain, the inventors proposed intraocular delivery of viral vectors to retinal cells, followed by administration of a DREADD agonist such as **1** [CNO] to engage downstream neural pathways. The central idea being that eye can serve as a clinically accessible entry point for influencing the photic regulation of arousal and mood (PRAM) pathway, which links the retina to the suprachiasmatic nucleus, dorsomedial hypothalamus, and locus coeruleus. Evidence from preclinical studies in rats suggests that intravitreal AAV delivery can drive DREADD expression in retinal cells and in retinal projections to the suprachiasmatic nucleus. Electrophysiology studies confirmed functional receptor engagement and utilizing inhibitory DREADD constructs, both systemic **1** [CNO] and tropical eye drop administration reduced electroretinogram amplitude, supporting the feasibility of ocular agonist delivery as a noninvasive activation route. Furthermore, when a constant-darkness model was used to induce depression-like phenotypes, activation of excitatory retinal DREADDs prevented darkness-associated weight-loss, improved saccharin preference, and reduced immobility in the forced-swim test, while leaving anxiety-like behavior and general locomotion largely unchanged. Together these data suggests that retinal chemogenetic stimulation can selectively attenuate depression-like outcomes without broadly disrupting other behavioral domains. Additionally, PACAP (pituitary adenylate cyclase-activating polypeptide) promoter was introduced to restrict DREADD expression to a melanopsin-positive retinal ganglion cell population. Findings from co-localization studies indicate that PACAP-driven constructs are expressed predominantly in melanopsin-immunoreactive cells, and activation of this specific neuronal population was sufficient to reduce depression-associated weight loss under constant-darkness conditions. Overall, the invention aims to activate specific neurological pathways in a physiologically benign and minimally invasive manner, thereby mitigating risks associated with intracranial interventions.

### Drexel University and the J. David Gladstone Institutes

Researchers from Drexel University and The J. David Gladstone Institutes introduced a cell-based regenerative strategy for neural repairs following nervous system injury (US2024325452A1) [146]. In this approach human pluripotent stem cells (hPSCs) derived neural progenitors, particularly V2a spinal interneurons, are engineered to express DREADDs so that their activity can be reversibly controlled after transplantation. The patent aims to address a longstanding limitation of neural transplantation where implanted cells typically act without direct post-engraftment control by modulating graft function on demand. DREADD-expressing V2a interneurons was generated from hPSCs following a stepwise differentiation protocol. Electrophysiological data show spontaneous neuronal firing, which is silenced by clozapine, consistent with successful inhibitory DREADD function. Furthermore, evidence show that transplanted human cells survive in the injured spinal cord and differentiate into neurons. Additionally, clozapine-mediated silencing of the graft reduces diaphragm electromyographic activity in a rat cervical spinal cord injury model, including during hypoxia challenge, suggesting functional integration with host respiratory circuitry. Together, these data highlight that transplanted neural cells can survive, mature, integrate, and then be pharmacologically tuned after engraftment. Overall, this combinatorial stem cell and chemogenetic platform offers promising therapeutic potential for currently untreatable neurological conditions.

### Assaf and Schiller (Individual Assignee)

Inventors Assaf and Schiller reported a chemogenetic strategy for treating abnormal basal ganglia signaling in movement disorders using DREADDs (WO2017153995A1) [147]. The inventors rationalize that hypokinetic disorders such as Parkinson’s disease may be improved by suppressing activity in the internal globus pallidus (GPi) and substantia nigra pars reticulata (SNr), while enhancing activity in the external globus pallidus (GPe), subthalamic nucleus (STN), and potentially the motor thalamus. AAV vectors were utilized to deliver inhibitory (hM4Di) and excitatory (hM3Dq) DREADDs in 6-hydroxydopamine mouse model of Parkinson’s disease and the motor outcomes were assessed by open-field behavior, turning bias, and rotarod performance. Findings from Parkinson’s disease model showed that inhibition of entopeduncular nucleus (EP)/GPi- equivalent and SNr with G_i_-DREADDs improved locomotor activity, reduced ipsilateral turning, and increased rotarod performance, consistent with lowering excessive basal ganglia output. Furthermore, activation of GPe with G_q_-DREADDs improved all major motor readouts, supporting the idea that dampening indirect-pathway overactivity can restore movement. More importantly, excitatory G_q_ activation of the STN produced robust and somewhat unexpected benefit: unilateral STN activation improved locomotion and turning, while bilateral STN activation produced large gains in both movement velocity and rotarod performance. Overall, the work highlights that targeting multiple nodes of the basal ganglia network, via combined suppression of GPi/SNr output together with activation of GPe and STN, may yield stronger restoration of motor function than single-site intervention alone.

### Ovid Therapeutics Inc

Matthew During of Ovid Therapeutics Inc. proposed a chemogenetics based precision neuromodulation strategy for treating epilepsy and related seizure disorders (WO2018170223A1) [148]. The central idea is to deliver engineered receptors via viral vector method to seizure-prone brain region to achieve targeted modulation of epileptogenic circuits in presence of an exogenously administered ligand. While the invention places particular emphasis on inhibitory DREADD-hM4Di, hM3Dq and KORD could also serve as alternative options. **1** [CNO] and **4** [perlapine] served as ligands for muscarinic DREADDs and **8** [SalB] for KORD. The proposed clinical scope is broad, spanning focal epilepsy, temporal lobe epilepsy, focal cortical dysplasia, status epilepticus, tumor-associated seizures, and numerous severe pediatric epileptic encephalopathies. A major technical contribution of this invention is the vector design framework. The application describes viral delivery systems, especially adeno-associated virus and lentiviral vectors, that encode modified receptors under neuronal promoters such as murine CaMKII or human CaMK2A, together with regulatory elements to support expression in target neurons. The dual-modality design allows fine-tuned regulations of neuronal networks implicated in seizure generation.

### National Institutes of Health and the Johns Hopkins University

**Fig. 5:**
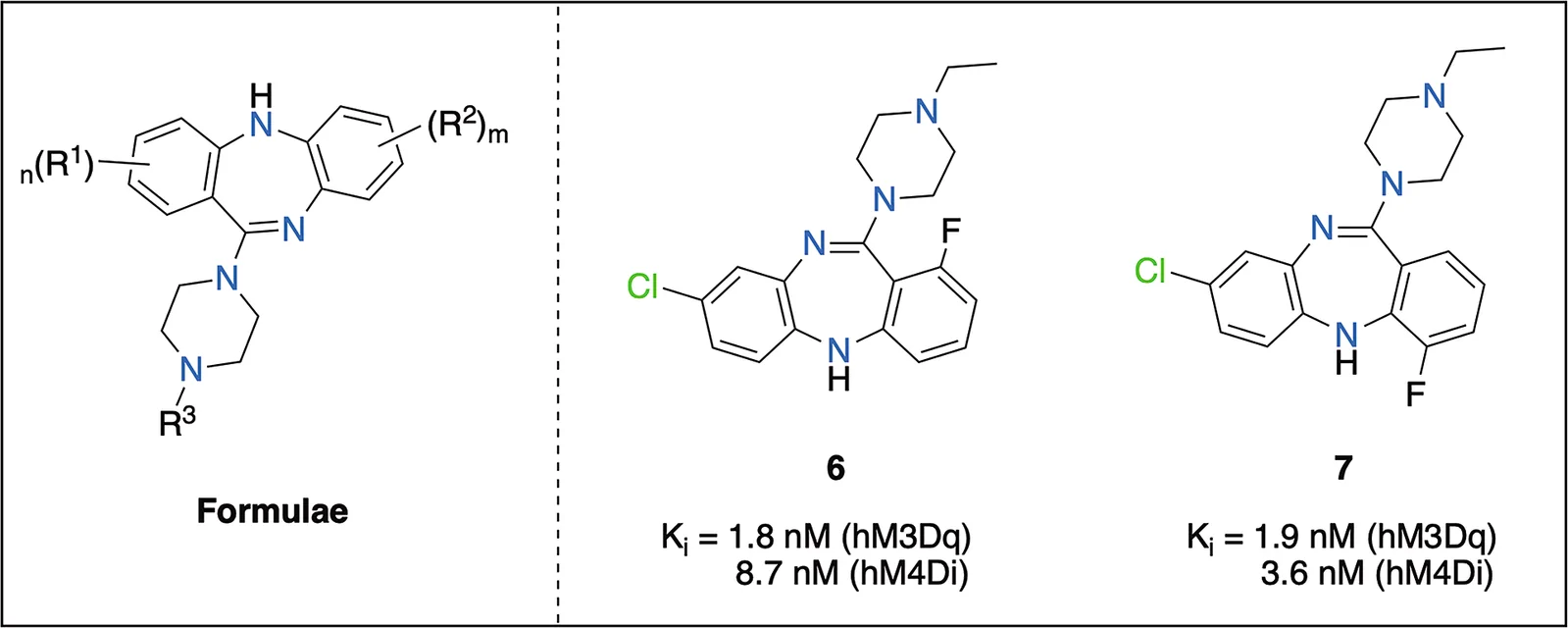
Structures covered by NIH and JHU for new DREADD activators

Researchers from the National Institutes of Health and the John Hopkins University reported a series of halogenated dibenzo[b, e][1,4]diazepine derivatives as next-generation ligands for DREADDs, particularly hM3Dq and hM4Di (WO2019157083A1) [149]. Within the disclosed series, **6** [JHU37152] and **7** [JHU37160] emerged as lead compounds with high binding affinities (Ki = 1.7 and 1.9 nM at hM3Dq; Ki = 8.7 and 3.6 nM at hM4Di, respectively). In addition, both compounds robustly activate DREADD signaling with sub-nanomolar hM4Di-mediated G_i/o_ responses and low nanomolar hM3Dq-mediated calcium responses. Studies from ex vivo autoradiography indicated that **7** [JHU37160] could selectively displace [^3^H]clozapine binding from DREADD sites at low concentration, supporting a favorable selectivity window. In in vivo studies conducted on transgenic mice, both ligands demonstrated significant DREADD-associated behavioral effects, and in the open-field studies suppressed locomotor activity more strongly than clozapine in D1-hM3Dq animals. The invention also discloses PET-compatible radiolabeled analogs. Among these, [^18^F]**11** prepared at > 95% radiochemical purity showed favorable pharmacokinetics and enabled direct visualization of hM4Di expression in the macaque amygdala and putative projection sites. Furthermore, these ligands find application in DREADD-assisted metabolic mapping (DREAMM). **7** [JHU37160] produced distinct, receptor-dependent metabolic network changes in D1-hM3Dq and D1-hM4Di mice without significant effects in wild-type mice. On the contrary, **2** [C21] altered brain metabolism in wild-type mice. Overall, this invention presents these ligands as dual-purpose chemogenetic tools-both as actuators and imaging agents, with improved pharmacological properties compared to **1** [CNO] and **2** [C21] (Fig. 5).

### Columbia University

Zuker and Jin at Columbia University reported a discrete vagal-brainstem circuit that senses peripheral inflammation and actively regulates systemic immune response (WO2023114892A2) [150]. Activation of the neurons in the caudal nucleus of the solitary tract (cNST) was achieved using lipopolysaccharide (LPS) as an innate immune system. Activity-dependent labeling and chemogenetic manipulation suggests that these LPS-responsive cNST neurons act as an endogenous anti-inflammatory break. Their activation suppresses pro-inflammatory cytokines such as TNFα and IL-6 while increasing the anti-inflammatory cytokine IL-10, whereas silencing leads to increased cytokine release and reduced anti-inflammatory output. Single-cell analyses map these immune-responsive brainstem neurons to a Crhr2-enriched cluster, and activation of this genetically defined population is reported to be sufficient to dampen inflammation. On the afferent side of the circuit, in vivo calcium imaging of the nodose ganglion identifies distinct vagal sensory neuron populations that respond selectively to pro-inflammatory versus anti-inflammatory cytokines. The patent associates pro-inflammatory signaling, including TNF-α responses, with Calca/CGRP-related vagal neurons and anti-inflammatory signaling with TRPA1-expressing vagal neurons. Importantly, chemogenetic activation of TRPA1-positive vagal neurons phenocopies cNST activation, lowering pro-inflammatory cytokines and increasing anti-inflammatory cytokines, suggesting that peripheral neural targets may provide access to central immune-control pathways.

### Howard Hughes Medical Institute

Sternson and Magnus at the Howard Hughes Medical Institute presented an elegant chemogenetic strategy for engineered ligand-gated ion channels (LGICs) with orthogonal pharmacology, enabling precise, cell type-specific control of ionic conductance [151, 152]. Using a “bump-hole” approach, the authors systematically mutated the α7 nicotinic acetylcholine receptor ligand-binding domain and screened a focused library of aminoquinuclidine benzamide ligands to identify matched pharmacologically selective actuator module (PSAM)-effector molecule (PSEM) pairs. The resulting chimeric channels, coupled to diverse ion pore domains, could selectively activate or silence genetically defined neurons without perturbing endogenous signaling. Critically, the system demonstrated robust in vivo efficacy: PSEM-mediated silencing of hypothalamic AGRP neurons effectively suppressed optogenetically evoked feeding behavior in mice. This modular, combinatorial framework represents a significant advance in chemogenetic neuroscience tooling, complementing existing optogenetic approaches with tunable ionic conductance control.

Later in 2019, the same group reported a significant advance in chemogenetic technology through the development of ultrapotent, ion channel-based tools for precise neuronal control [153]. By engineering mutations in the α7 nicotinic acetylcholine receptor ligand-binding domain, the authors created chimeric channels-PSAM4-GlyR and PSAM4-5HT3-capable of silencing or activating neurons in response to the FDA-approved smoking cessation drug varenicline at sub-clinical doses. They further synthesized a library of highly selective agonists, termed uPSEMs, with sub-nanomolar potency and minimal off-target activity. Efficacy was validated across multiple modalities-electrophysiology, calcium imaging, behavioral assays, and PET imaging-in both rodent and non-human primate models. This platform addresses longstanding limitations of existing chemogenetic systems and offers a promising translational pathway toward human therapeutic applications in neurological disorders.

Sternson and Magnus further described a novel chemogenetic strategy to treat substance use disorders (WO2023212230A1) [81]. The central idea involves coupling drug exposure directly to activity changes in selected neural circuits so that modulation occurs only during drug intake and pharmacokinetics of the abused substance can be tracked. The invention primarily focuses on engineered receptors built from mutated α7 nicotinic acetylcholine receptor ligand-binding domains fused to either 5-HT3 ion pore domains for excitation or GlyR pore domains for inhibition. Using a high-throughput discovery pipeline, 277 mutated chimeric ion channels were screened against 21 compounds to identify receptors activated by addictive drugs but less responsive to endogenous agonists. Further mutation yielded two lead receptors: coca-5HT3, an excitatory receptor and coca-GlyR, an inhibitory receptor which are selective for cocaine over major cocaine metabolites and several other addictive drugs. In neuronal assays, coca-5HT3 produced sustained depolarization and action potential firing, whereas coca-GlyR reduced excitability, lowered input resistance, and increased rheobase without materially altering baseline membrane properties. In rat models, viral expression of coca-5HT3 in the lateral habenula-a brain region naturally inhibited by cocaine-reversed this inhibition, suppressing cocaine self-administration and blunting cocaine-induced dopamine elevation in the nucleus accumbens, as confirmed by PET imaging and fiber photometry. Crucially, food motivation and natural reward-seeking remained unaffected [154]. This closed-loop chemogenetic strategy represents a promising foundation for developing highly targeted gene therapies for cocaine use disorder.

### Broad Institute and Massachusetts Institute of Technology

Feng, Roy and Zhang from the Broad Institute and Massachusetts Institute of Technology proposed a targeted chemogenetic strategy to ameliorate cognitive deficits associated with neuropsychiatric disorders (WO2023278295A1) [155]. This patent positions the relatively understudied anterodorsal (AD) thalamus as a convergence node for cognitive dysfunction across genetically distinct neuropsychiatric models. Using circuit-specific CRISPR knockdown in mice, the inventors report that multiple autism- and schizophrenia-associated genes, including PTCHD1, YWHAG, GRIA3, CACNA1G, HERC1, CNTNAP2, MTOR, and ATP1A3, are prominently represented in the anterior thalamic region. Knockdown of several of these risk genes in the anterodorsal (AD) thalamus is associated with neuronal hyperexcitability and cognitive impairment. AAV-delivered inhibitory DREADD hM4Di upon activation with **2** [C21] normalized AD thalamus function, reduced hyperexcitability, and improved cognitive performance in preclinical models. Overall, these findings underscore the therapeutic potential of chemogenetic silencing in restoring cognitive function across a diverse range of neuropsychiatric disorders.

### Gateway Biotechnology, Inc

Bao and Pinkl at Gateway Biotechnology, Inc. disclosed a chemogenetic-based therapeutic platform in which adeno-associated viral (AAV) vectors paired with neuron-selective promoters, are used to deliver DREADDs to a defined neural population. The approach is designed to enable pharmacologic control of inhibitory or excitatory neuronal activity after administration of a small molecule actuator such as **1** [CNO]. Although, the claims extend broadly across neurological disorders, the patent focuses on auditory circuitry and proposes applications in tinnitus, Meniere’s disease, noise-induced hearing loss, sensorineural hearing loss, auditory neuropathy spectrum disorder, and central auditory processing disorder (WO2024182608A2) [156]. Target regions include the inferior colliculus, auditory cortex, hippocampus, medial geniculate body, amygdala, and caudate nucleus, with promoter options such as CaMKII, hSyn, and VGAT (Vesicular GABA Transporter) to improve cell-type specificity. In vivo studies conducted in mouse, demonstrated that chemogenetic activation of excitatory neurons in the inferior colliculus can drive tinnitus-like behavior. In this model, bilateral stereotaxic delivery of an excitatory DREADD construct was followed by behavioral testing in a sound-based avoidance paradigm. After **1** [CNO] administration, majority of the mice exhibited tinnitus-positive behavior, whereas saline injections and **1** [CNO] administration in control animals did not produce the same effect. Collectively, this work established a compelling proof-of-concept for chemogenetic modulation of auditory circuitry, positioning the AAV-DREADD platform as a promising and highly programmable therapeutic strategy for disorders in which aberrant neuronal activity underlies the pathology.

## Recent advances and future prospects

For clinical translation and to be fully effective within CNS, an ideal chemogenetics system must satisfy several criteria as succinctly outlined by Magnus et al. [153]. The agonist should be a safe, well-tolerated, and a drug capable of crossing the blood-brain barrier. The DREADD agonist must exhibit high selectivity for the engineered receptor, avoiding off-target interactions with endogenous receptors. Despite their dominance in neuroscience research, DREADDs have yet to cross the threshold into clinical application. The absence of significant ‘chemogenetics’ clinical trials except for smaller trials in various stages of patient recruitment (#NCT07085195, #NCT06596681, #NCT07533591) in epilepsy, Parkinson’s disease and refractory neuropathic pain have been initiated in China as reported on ClinicalTrials.go. In comparision, hundreds of trials are listed for traditional drugs like clozapine—underscores a critical lack of available ligands. This disparity is particularly glaring because chemogenetics offers a level of targeted, reversible control that could surpass current therapies. Significant progress in ligand development will accelerate to bridge the gap from bench to bedside. A recent innovation is the development of a DREADD based on the hydroxycarboxylic acid receptor 2 (HCA2) termed HCAD. This receptor is predominantly expressed in immune cells with little to no central expression and is involved in lipolysis, inflammation, and insulin sensitivity. HCAD and its ligand will allow for selective modulation of immune-metabolic pathways without affecting central nervous system function. Activation of HCAD in macrophages or adipose tissue can modulate inflammation-related insulin resistance and how precise immunometabolic signaling affects metabolic syndrome and type 2 diabetes [70].

Immunogenicity remains a significant challenge for DREADD viral vector delivery, especially when using adeno-associated virus (AAV) and lentiviral vectors [92]. Both innate and adaptive immune responses can impact the safety, efficacy, and durability of transgene expression in gene manipulation experiments. Innate immune response can be altered upon administration of AAVs, the body’s innate immune system—via pattern recognition receptors (PRRs) like Toll-like receptors (TLRs)—can recognize and activate inflammatory pathways in response to AAV or lentiviral DNA or RNA. Activation of these pathways can lead to cytokine release, inflammation, and tissue damage, limiting therapeutic effect and potentially resulting in neurotoxicity, especially in the CNS. Similarly the adaptive immune system may generate neutralizing antibodies against the viral capsid and/or the transgene product. Pre-existing immunity, especially to common AAV serotypes, is widespread in humans, reducing vector efficacy and raising safety concerns for repeat administration. CD8 + T cells can destroy transduced cells presenting AAV or lentivirus peptides, leading to loss of transgene expression and therapeutic effect. High doses or long-term, stable DREADD expression may provoke stronger immune responses, resulting in cell death signatures, neurotoxicity, or loss of effect. Promoters, serotypes, and payload sizes can alter immune activation markers and exacerbate inflammation. CNS-targeted AAV delivery can trigger DNA damage response (DDR)-dependent innate immune signaling, leading to pro-inflammatory cytokine production, astrocyte activation, and neuronal toxicity. Further modulation of signaling pathways like STING and p53 shows promise in mitigating excessive CNS immune activation without fully suppressing vector activity. These approaches have not yet gained widespread acceptance. Lentiviral vectors, while able to package larger constructs and deliver stable expression, pose additional risks of genome integration and trigger both transient innate and adaptive immunity, usually through recognition of lentiviral RNA and reverse-transcribed DNA, activating TLR3 and TLR7. Pro-inflammatory cytokines released after lentiviral administration can limit cell transduction efficacy and prime T cells against the vector and transgene product.

The structural biology of DREADD ligand have mainly been studied in its active form (agonist/ G protein / miniG). As noted earlier, most structures so far are active (agonist + G protein / miniG). It would be valuable to design ligands and obtain high-resolution structures of DREADDs in inactive / antagonist bound states of the receptor without G protein (or with inverse agonist), or bound to antagonists, to see conformational constraints, baseline (unstimulated) states, etc. Design and evaluation of partial agonist and/or biased ligands along with allosteric modulators may further add new dimensions to the utility of DREADDs. Alternatively, the role of cryptic pockets and Na + ion modulation leading to differential ligand effects may be of interest. With the availability of numerous GPCR ligand bound active and inactive state structures, chemogenetic versions of other GPCR families, engineered for specific G protein coupling, etc. is now theoretically possible. Engineered receptors for new GPCRs could form the next frontier in DREADDs research. Structures of *mini-DREADDs* or truncated variants (e.g. miniDq / miniDi) that have been functionally characterized would allow for discerning the conformation dynamics and differences of their full-length receptors. Conformational ensemble work using methods like Hydrogen-Deuterium Exchange (HDX), Nuclear Magnetic Resonance (NMR), Fluorescence Resonance Energy Transfer (FRET), Double Electron-Electron Resonance (DEER), or time-resolved cryo-EM could further illuminate the DREADDs kinetics and dynamics. Use of nanodiscs, peptidiscs, or styrene-maleic acid lipid particles (SMALPs) to reconstitute DREADDs in lipid bilayers close to native conditions for structural and functional fidelity. For robust in situ information, cryo-electron tomography (cryo-ET) could be applied to visualizing engineered GPCRs within membrane environments. Unlike permanent genetic knockouts, DREADDs allow for preserving developmental pathways, enabling the study of adult-onset interventions. Utilization of humanized DREADDs is an area where DREADDs can offer a viable pipeline for realizing the translation potential of this chemogenetic technology. Design and development of peripherally acting ligands will further expand the use in CNS sparing therapies.

Beyond static structures, molecular dynamics simulations have provided insights into DREADD microstate transitions and G protein coupling mechanisms. These methods complement cryo-EM, revealing transient conformations and energetically favored ligand orientations within the binding site. Moreover, AI-assisted cryo-EM fitting approaches proposed could integrate AlphaFold-like predictions with MD-guided density fitting, allowing higher fidelity modeling of ligand–receptor complexes at atomic detail for systems including GPCRs and DREADDs. A significant issue associated with DREADD technology is the potential for receptor desensitization and downregulation following repeated activation by chemical actuators. Repeated dosing of a DREADD ligand can lead to reduced responsiveness, as the receptors undergo desensitization and subsequent internalization and downregulation. Detailed studies in higher species deconvoluting the dosing regimens may thus need to be carried out before the translational potential of this chemogenetic approach can be realized. In summary with the promise of ‘omics’ approaches in combination with machine learning explorations, the chemogenetic DREADDs technology is primed to take a huge leap towards precision mechanism-informed, and personalized neuromodulation in both preclinical models and future clinical applications.

## Data Availability

No datasets were generated or analysed during the current study.
